# Phytochemical and Bioactivity Profiling of Unconventional Food Plant, *Boehmeria caudata* Leaves: FT‐IR, GC–MS, Experimental, and In Silico Investigation

**DOI:** 10.1002/fsn3.70953

**Published:** 2025-09-14

**Authors:** Md. Liakot Ali, Md. Ajib Jaber, Zihanul Hasan, Nawreen Monir Proma, Neamul Hoque, Md. Tashrif Rahman Tipu, Kutub Uddin Ahamed, Bakul Akter, Bibi Humayra Khanam, Mohammed Kamrul Hossain

**Affiliations:** ^1^ Department of Pharmacy Faculty of Biological Sciences, University of Chittagong Chittagong Bangladesh; ^2^ Pharmaceutical Sciences Research Division BCSIR Dhaka Laboratories, Bangladesh Council of Scientific and Industrial Research (BCSIR) Dhaka Bangladesh

**Keywords:** *Boehmeria caudata*, functional foods, in vivo, neuropharmacological, unconventional food plant, vitamin E

## Abstract

*Boehmeria caudata* (Urticaceae) is an unconventional food plant found extensively across the neotropical region, including Central and South America, where its leaves are consumed as vegetables and traditionally used to treat sore throats. This study aimed to analyze the phytochemical composition of the methanolic extract of *B. caudata* leaves (MEBCL) and explore its pharmacological effects through in vitro*,* in vivo*,* and *in silico* methods. GC–MS investigation identified 14 phytochemicals, including two forms of Vitamin E. MEBCL exhibited a strong in vitro antioxidant effect, with IC_50_ values of 63.61 μg/mL for the DPPH assay as well as 130.66 μg/mL for the FRAP assay. In vivo, MEBCL showed notable analgesic effects, as observed in both the acetic acid‐induced writhing model and the formalin‐induced paw‐licking model. MEBCL also showed significant anxiolytic actions in the elevated plus maze and hole board tests. Additionally, MEBCL significantly reduced immobility time in both the forced swimming as well as tail suspension tests, indicating its antidepressant properties. Molecular docking studies revealed that the phytochemicals in MEBCL exhibited significant binding affinity to key drug target proteins associated with oxidative stress, pain, anxiety, and depression. These compounds also have a favorable pharmacokinetic and safety profile, suggesting their potential as future drug candidates. Therefore, MEBCL can be a valuable reservoir of Vitamin E with notable antioxidant, analgesic, anxiolytic, and antidepressant effects. However, more in‐depth studies are essential to determine whether *B. caudata* leaves can be effectively used as a functional food and therapeutic agent.

## Graphical abstract

In this paper, we have investigated the in vitro antioxidant as well as in vivo analgesic, anxiolytic, and antidepressant activities of the methanolic extract of Boehmeria caudata leaves, alongside a phytochemical evaluation using FT‐IR and GC‐MS analysis. Additionally, molecular docking and ADMET studies were conducted to understand the possible mechanisms of action and pharmacokinetic profiles of the compounds identified from this plant.

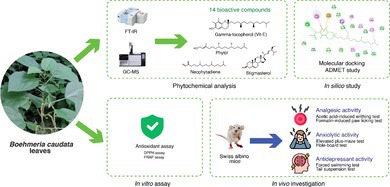

## 1 Introduction

Pain is a sensation that is linked to mental suffering (Perl 2011) that affects approximately 20% of adults worldwide, with 10% of new cases of chronic pain diagnosed each year. Pain is the leading reason for more than 80% of doctor visits, with most cases being brief and easily managed. However, for some people, the pain lingers, turning into a persistent struggle and a continuous source of discomfort (Ayanaw et al. 2023). Anxiety is another common condition that often occurs alongside depression or other health issues. Globally, one in five individuals will experience an anxiety disorder at some point in their lives (Garber and Weersing 2010). Numerous studies suggest that anxiety and depression often occur together rather than as separate conditions. Approximately one‐third of individuals diagnosed with depression also experience anxiety disorders (Hossen et al. 2021). Nonsteroidal anti‐inflammatory drugs (NSAIDs) continue to be the mainstay of medication for pain relief. However, their prolonged use is limited due to adverse effects, particularly nephrotoxicity (Sany et al. 2025). To tackle depressive disorders as well as anxiety, several drug classes include the γ‐aminobutyric acid (GABA)ergic, serotonergic, and glutamatergic pathways. Medications such as benzodiazepines, barbiturates, and selective serotonin reuptake inhibitors (SSRIs) are usually recommended. However, these medications can cause side effects, including migraines, sexual dysfunction, dependence, seizures, and, in some cases, an increased risk of suicidal thoughts (Ali, Meem, et al. 2024). Hence, there is still a pressing need for bioactive compounds that provide greater therapeutic benefits while minimizing side effects. Medicinal plants have historically been a valuable reservoir of treatments for many diseases, including the common cold and cancer (Ali, Noushin, et al. 2024). Therefore, systematic exploration, particularly of lesser‐studied species, is of great interest for identifying safer, more effective, and cost‐efficient pharmaceutical alternatives.

Unconventional food plants (UFPs), also referred to as nonconventional food plants, are species with edible parts that are not commonly consumed in daily diets. These plants often require unique processing methods and typically lack significant market presence, being either absent from commercial markets or sold on a limited scale (Milião et al. 2022). The growing global population has driven the need for alternative food sources that align with evolving dietary trends, emphasizing nutrition and health‐conscious choices. As a result, interest in UFPs has risen due to their rich nutritional profile, providing essential macronutrients, micronutrients, and bioactive compounds with health‐promoting effects (Ferreira Júnior et al. 2021; Milião et al. 2022). Over the past decade, the consumption of UFPs has increased, as they naturally grow without chemical additives and, in some cases, offer superior nutritional benefits compared to commonly consumed crops (Azam et al. 2014). Additionally, certain UFPs have been traditionally utilized to treat a wide variety of diseases, including wound healing and the treatment of infectious and inflammatory diseases. Plants classified as UFPs, for example, *Moringa oleifera*, *Jatropha curcas* , *Pereskia aculeata* , and *Gramineae bambusoideae*, stand out as promising alternatives for food system applications. They not only surpass traditional staples like rice and wheat in protein concentration (on a dry basis) but also provide a balanced array of essential amino acids and appear to contain health‐promoting bioactive peptides (Milião et al. 2022). However, research on their bioactive properties remains limited. Given their dual role in nutrition and medicine, these plants have attracted significant interest from both the food and pharmaceutical industries (Peisino et al. 2020).

Genus *Boehmeria* (Urticaceae) encompasses small trees, shrubs, and perennial herbs, and is distinguished from other genera of the family by the absence of stinging hairs, hence its common names “falsenettle” and “ramie.” Its roots, leaves, and flowers have long been used in folk medicine for antioxidant, antipyretic, diuretic, hemostatic, anti‐inflammatory, and hepatoprotective purposes (Chung and Shin 1998; Kim et al. 2006). Notably, Akter et al. highlighted that Boehmeria species are rich in active constituents such as rutin, isoquercetin, kaempferol‐3‐O‐rutinoside, and (−)‐cryptopleurine—compounds widely recognized for their roles in managing various diseases (Akter et al. 2018). *Boehmeria caudata*, an unconventional food plant, is widely distributed across the neotropical region, spanning Central and South America. In these areas, it is commonly referred to as “lixa‐da‐folha‐larga”, “urtiga‐mansa”, “assa‐peixe”, and “folha‐de‐santana” (de Paiva et al. 2020). In Brazil, the leaves of this plant are commonly eaten as a vegetable. Traditionally, their decoction has been used as a remedy for sore throats and skin boils (da Cruz Alves et al. 2024; Yazbek et al. 2019). Existing literature indicates that the aerial parts of this plant exhibit chemotherapeutic and anti‐inflammatory properties (de Paiva, Nunes, et al. 2020). However, a thorough investigation of the phytochemical and pharmacological aspects of its leaves is needed to better assess its true potential as both a nutritional and medicinal source.

This investigation aims to identify phytochemicals present in the methanolic extract of *B. caudata* leaves (MEBCL) using FT‐IR as well as GC–MS analysis. Additionally, the in vitro antioxidant activity as well as in vivo analgesic, anxiolytic, and antidepressant effects of MEBCL were assessed. Furthermore, in silico molecular docking and ADMET analysis were conducted to investigate potential mechanisms of action and pharmacokinetic properties.

## 2 Materials and Methods

### 2.1 Chemicals and Reagents

Acetic acid, formalin, DPPH, acetate buffer, TPTZ solution, FeCl_3_ solution, and Tween‐80 were procured from Nurjahan Scientific Limited, Chittagong, Bangladesh. The standard drugs, including diazepam, diclofenac sodium, and fluoxetine, were generously provided by Albion Laboratories Limited, Chittagong, Bangladesh.

### 2.2 Collection of Plant Material and Extract Preparation

*B. caudata* leaves were obtained from the south campus of the University of Chittagong, Chittagong, Bangladesh, and identified by Dr. Shaikh Bokhtear Uddin, Professor of Botany at the University of Chittagong, with the voucher specimen number CU/Pharm 1256. Collected plant samples were cleaned with water and then dried for 10 days in a room with adequate airflow, but not directly exposed to sunlight. The dried samples were powdered using a blender. A total of 400 g of the powdered leaves was stored in amber‐colored glass containers and macerated in 4.5 L of methanol for 2 weeks. The containers were occasionally shaken to facilitate proper extraction. After 2 weeks, the mixture was filtered. To obtain a concentrated extract, methanol was evaporated using a rotary evaporator. The resulting crude extract (MEBCL) weighed 21 g and was stored at 4°C for future analysis.

In this investigation, the choice of methanol as a solvent is based on its frequent application in extraction procedures and its proven ability to efficiently extract bioactive constituents, particularly phenols and flavonoids with important pharmacological relevance. Owing to its polarity and versatile solubility, it can dissolve and recover compounds of both polar and nonpolar nature (Islam et al. 2025).

### 2.3 Fourier Transform‐Infrared (FT‐IR) Spectroscopy and Gas Chromatography–Mass Spectrometry (GC–MS) Analysis

MEBCL was analyzed using FT‐IR spectroscopy and GC–MS following standard operating procedures. The detailed experimental methods are available in the Supporting Information.

### 2.4 In Vitro Antioxidant Assay

The antioxidant potential of MEBCL was evaluated in vitro using two well‐established methods: the 1,1‐diphenyl‐1‐picrylhydrazyl (DPPH) free radical scavenging assay and the ferric reducing antioxidant power (FRAP) assay, following the protocol described by Anjum et al. (Singh et al. 2019). Ascorbic acid was utilized as the reference standard for both tests.

### 2.5 Test Animals and Ethical Approval

Swiss albino mice (gender: male), weighing between 20 and 30 g, were obtained from the animal house at the University of Chittagong. They were kept in clean, dry cages in a regulated setting with a cycle of 12 h of light and darkness. The mice had full freedom to consume a standard meal as well as water. They were subjected to a 1‐week acclimatization period, with food withdrawn for 12 h prior to and during the experiments. The Animal Ethics Review Board (AERB), Faculty of Biological Sciences, University of Chittagong, approved this study using approval form number AERB‐FBSCU‐20250622‐(1).

### 2.6 Experimental Design

The animals were divided into four groups. The negative control group received only the vehicle, 1% Tween‐80 solution, which was used to dilute the plant extract and prepare the doses. The positive control group was treated with a standard drug, while two experimental groups (*n* = 5) were given MEBCL at doses of 200 mg/kg and 400 mg/kg, respectively. The analgesic activity was compared against diclofenac sodium, whereas diazepam and imipramine were employed as standard drugs for assessing anxiolytic and antidepressant effects, respectively. All treatments were administered orally.

### 2.7 Acute Oral Toxicity Test

The acute oral toxicity of MEBCL was evaluated following OECD guidelines (Sany et al. 2025). A total of six doses of MEBCL (100, 250, 500, 750, 1000, and 2000 mg/kg) were used for this investigation. Mice (*n* = 3, gender: male) received the extract at the aforementioned doses for 14 days, and any signs of toxicity were documented. Observations were conducted during the first hour, then hourly for the next 4 h, and subsequently every 24 h over 14 days. A dose equivalent to 10% of the LD50 was regarded as safe.

### 2.8 Evaluation of Analgesic Effects

#### 2.8.1 Acetic Acid‐Induced Writhing Test

Pain was induced via an intraperitoneal injection of 0.7% glacial acetic acid at a dose of 10 mL/kg. The mice were categorized into groups and treated as per the protocol detailed in section “2.6. Experimental Design”. The standard control group received pretreatment 30 min before acetic acid administration, while the test groups were pretreated 60 min earlier. Writhing or squirming movements were observed for 20 min, starting 5 min post‐injection (Sany et al. 2025). The analgesic effect was determined by calculating the percentage inhibition of abdominal writhing using this formula:

$$\%\text{of inhibition}=\frac{A-B}{A}\times 100$$

Here, A and B are the number of writhing in the control group as well as the test groups, respectively.

#### 2.8.2 Formalin‐Induced Paw Licking Test

Each mouse received a subcutaneous injection of 20 μL of 1% formalin into their right hind paw, 60 min after MEBCL and diclofenac sodium treatment. Mice were grouped and treated following the guidelines in section “2.6. Experimental Design”. Pain response was assessed by recording the duration (in seconds) of licking and biting behavior in two phases: the initial neurogenic phase (0–5 min) as well as the late inflammatory phase (15–30 min) post‐formalin injection (Sany et al. 2025). The percentage of pain inhibition was calculated using the designated formula:

$$\begin{array}{c} \%\text{of pain inhibition}\\ =\frac{\text{Difference between reaction time of control group and treatment group}}{\text{Reaction time of control group}}\\ \times 100 \end{array}$$

### 2.9 Assessment of Anxiolytic Effects

#### 2.9.1 Elevated Plus‐Maze Test

For the elevated plus‐maze (EPM) test, an apparatus was used that consists of two open arms (30 × 5 × 0.2 cm) and two enclosed arms (30 × 5 × 15 cm^3^), all extending from a central platform (5 × 5 cm), 45 cm above the ground. The arms are positioned at right angles to each other. Mice were assigned to groups and treated as outlined in the “2.6. Experimental Design” section. Each mouse was positioned in the middle of the maze and directed to an open arm, following a 60‐min treatment period. The duration of time spent in the open and closed arms, the number of entries into each arm, and the preference for either arm were measured over a 5‐min testing period (Ali, Meem, et al. 2024).

#### 2.9.2 Hole‐Board Test

In conjunction with the EPM, the hole‐board test (HBT) is used to assess anxiety as well as stress responses in rodents. The test consists of a 40 × 40 × 25 cm^3^ wooden chamber with 16 evenly spaced 3 cm holes in the floor, elevated 25 cm off the ground to allow the mice to poke their heads through the holes. Mice were grouped and treated following the protocol in the “2.6. Experimental Design” section. Animals in an anxious state typically avoid exploring new areas, resulting in fewer head dips, whereas an increase in head dipping reflects curiosity and exploratory behavior. Mice were placed on the board 60 min after treatment and allowed to examine for 5 min, with the number of head dips recorded during the observation period (Ali, Meem, et al. 2024).

### 2.10 Assessment of Anti‐Depressant Effects

#### 2.10.1 Forced Swimming Test (FST)

For this test, mice were grouped and treated as described in the “2.6. Experimental animals” section. Sixty minutes after the treatment, each mouse was individually placed in a plastic container (25 × 15 × 25 cm^3^, 15 cm deep, with water maintained at 25°C ± 2°C). In the experiment, mice were kept from contacting the cylinder's bottom or jumping out. The time spent immobile—characterized by floating with minimal motion and maintaining the head above water—along with active swimming behavior was documented. The test lasted for 6 min, with the first 2 min used for acclimatization, and the subsequent 4 min measuring the time the mice spent immobile (Ali, Meem, et al. 2024).

#### 2.10.2 Tail Suspension Test (TST)

Mice were grouped and treated following the instructions in the “2.6. Experimental animals” section. Sixty‐min post‐treatment, each mouse was suspended by its tail 50 cm above the ground using adhesive tape placed about 1 cm from the tail's end. Antidepressant agents are anticipated to decrease immobility duration. In this experiment, each animal was suspended by its tail from a retort stand for 6 min, with the first minute allocated for acclimatization and the subsequent 5 min used to record immobility time (Ali, Meem, et al. 2024).

### 2.11 In Silico Investigation

#### 2.11.1 Protein Preparation

A total of four drug‐target proteins were selected (Table 1). The three‐dimensional structures of these proteins were retrieved from the Protein Data Bank (https://www.rcsb.org). The structures were then cleaned using Discovery Studio Visualizer software, eliminating any unnecessary atoms. Following this, the structures underwent hydrogen addition and energy minimization with SwissPDB Viewer (Guex and Peitsch 1997). The final optimized protein structures were prepared for molecular docking.

**TABLE 1:** Selected drug‐target proteins for investigating the pharmacological potentials of phytochemicals from *Boehmeria caudata*.

Pharmacological activity	Protein name	PDB ID
Antioxidant activity	Human erythrocyte catalase	1DGH
Analgesic activity	Human cyclooxygenase 2 (COX‐2)	5IKR
Anxiolytic activity	Human GABA_A_ receptor α1‐β2‐γ2 subtype	6X3W
Antidepressant activity	Human serotonin transporter	5I6X

#### 2.11.2 Ligand Preparation

The GC–MS analysis of the methanolic extract of *B. caudata* identified a total of 14 phytochemicals. These identified compounds were selected and downloaded in 3D SDF format from the PubChem database. For compounds available only in 2D, Open Babel GUI (O'Boyle et al. 2011) was used to convert them to 3D structures.

#### 2.11.3 Molecular Docking

The protein and ligand structures were uploaded into the PyRx software suite (Dallakyan and Olson 2015) and converted to PDBQT format using the Open Babel tool. A grid box of dimensions (25 × 25 × 25) Å was centered on the active site of each protein. A semi‐flexible docking approach was employed, keeping the protein rigid while allowing the ligands to be flexible. After docking, the pose with the lowest binding energy was selected and visualized using Discovery Studio Visualizer software (Studio 2008).

#### 2.11.4 ADMET Investigations

SwissADME server (Daina et al. 2017) was utilized to assess the Lipinski rule of five characteristics, whereas the pharmacokinetic and toxicological properties of MEBCL‐derived compounds were predicted using Deep‐PK, an advanced deep learning‐based online tool (Myung et al. 2024).

### 2.12 Statistical Analysis

All results were reported as mean ± standard error of the mean (SEM). One‐way ANOVA followed by Dunnett's multiple comparison test was used for statistical analysis using SPSS Version 16.0 (IBM Corp., NY). Statistical differences from the control group were considered significant at *p < 0.05, **p < 0.01, and ***p < 0.001.

## 3 Results

### 3.1 Fourier Transform‐Infrared (FT‐IR) Spectroscopy Analysis

**FIGURE 1:**
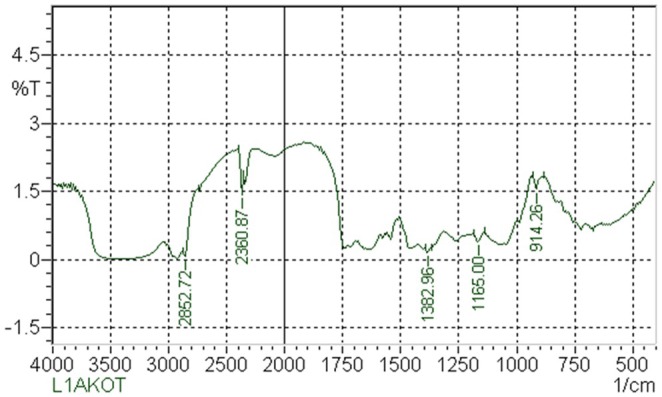
FT‐IR spectrum of methanolic extract of *B. caudata* leaves.

The FT‐IR analysis of MEBCL identified the presence of several important functional groups. The spectrum (Figure 1) showed distinct peaks indicating the presence of hydroxyl groups (O—H), likely from alcohols, carboxylic acids, and phenolic compounds. Additionally, carbonyl groups (C=O) were observed, suggesting the presence of aldehydes, carboxylic acids, esters, and amides. The presence of alkane (C—C), alkene (C=C), and alkyne (C≡C) groups further supports the existence of diverse organic compounds (Table 2). Moreover, the spectrum indicated the presence of amine (N—H) groups, ethers, and aromatic structures. These functional groups are indicative of various phytochemicals such as flavonoids, terpenoids, alkaloids, polyphenols, and tannins, highlighting the complex chemical profile of the plant extract.

**TABLE 2:** FT‐IR spectral data and characterization of phytochemicals in methanolic extract of *B. caudata* leaves.

Absorption (cm^−1^)	Peak intensity	Vibration mode	Characteristics group	Possible phytochemicals
914.26	1.5289	C—H bending	Alkane (C—H bending)	Alkane, aliphatic compounds
1165.00	0.3865	C—O stretching	Ether/Alcohol (C—O stretching)	Flavonoids, glycosides, tannins
1382.96	0.1558	C—H bending	Methyl/CH2 bending	Aliphatic compounds, saponins
2360.87	1.4231	C≡C stretching	Alkyne (C≡C stretching)	Alkynes, aromatic compounds (if associated)
2852.72	0.0719	C—H stretching	Alkane (C—H stretching)	Alkane, aliphatic compounds

### 3.2 Gas Chromatography–Mass Spectroscopy Analysis

**FIGURE 2:**
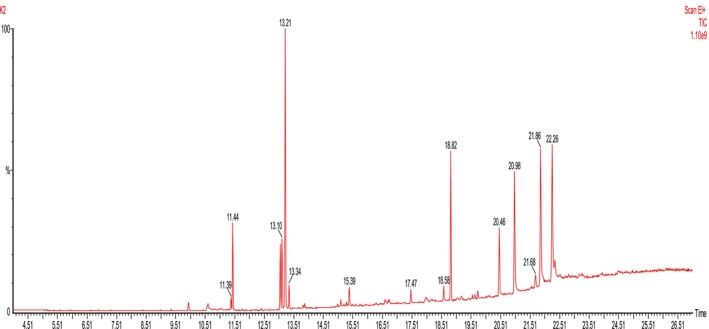
GC–MS chromatogram of methanolic extract of *B. caudata* leaves.

**FIGURE 3:**
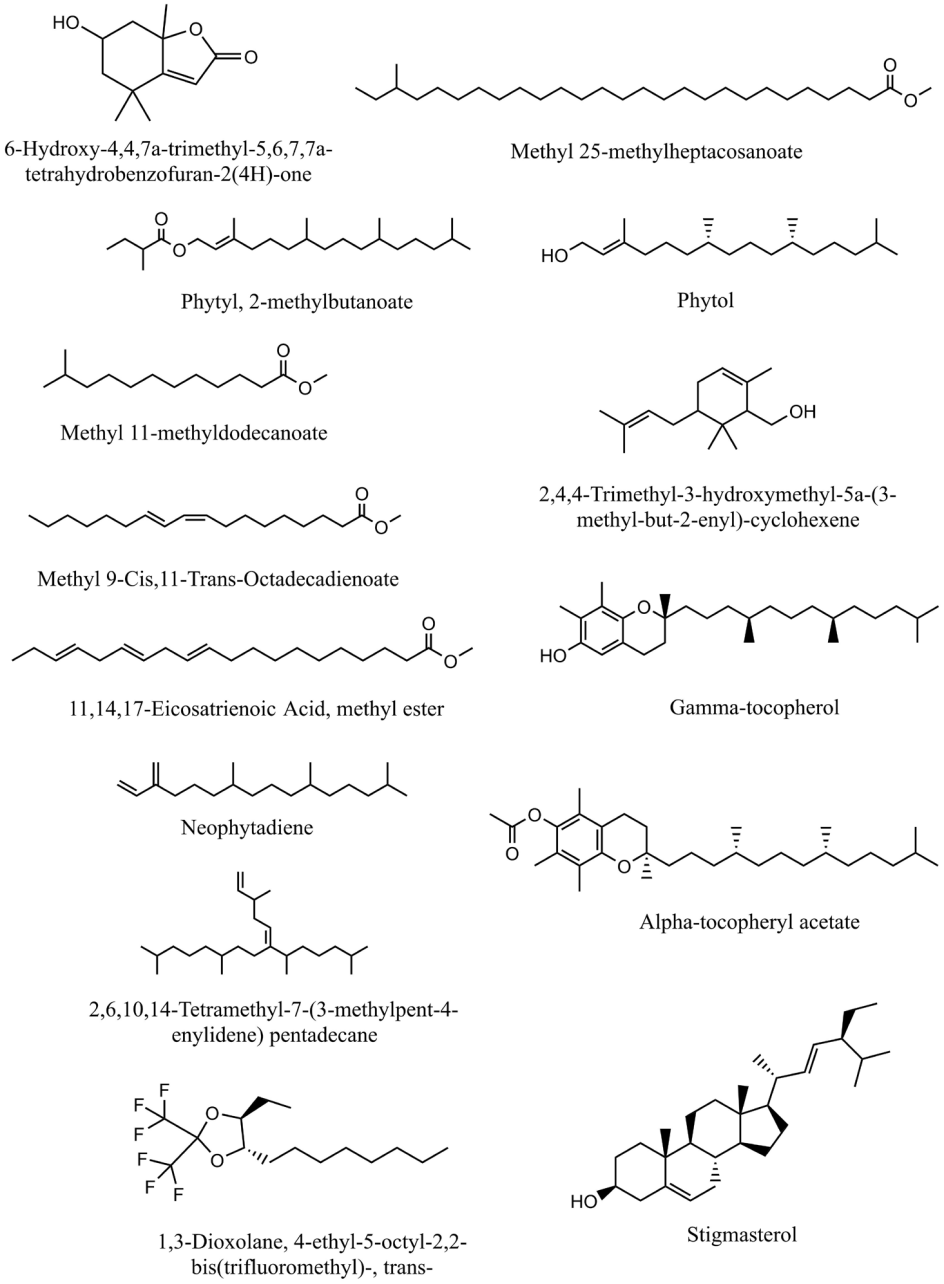
2D representations of the compounds identified in the methanolic extract of *B. caudata* leaves via GC–MS analysis.

The GC–MS analysis of MEBCL identified a total of 14 compounds. Figures 2 and 3 display the GC–MS chromatograms as well as the chemical structures of the identified compounds, respectively, while Table 3 provides detailed information on these compounds.

**TABLE 3:** GC–MS analysis of methanolic extract of *B. caudata* leaves.

Name	M.W.	Formula	RT	Similarity
6‐hydroxy‐4,4,7a‐trimethyl‐5,6,7,7a‐tetrahydrobenzofuran‐2(4H)‐one	196	C_11_H_16_O_3_	9.93	85.7
Methyl 25‐methylheptacosanoate	438	C_29_H_58_O_2_	10.42	90.4
Phytyl, 2‐methylbutanoate	380	C_25_H_48_O_2_	10.61	87.1
Neophytadiene	278	C_20_H_38_	10.82	84.5
Methyl 11‐methyldodecanoate	228	C_14_H_28_O_2_	11.44	90.0
11,14,17‐eicosatrienoic acid, methyl ester	320	C_21_H_36_O_2_	13.10	95.3
Methyl 9‐cis,11‐trans‐octadecadienoate	294	C_19_H_34_O_2_	13.21	97.2
Phytol	296	C_20_H_40_O	13.21	91.7
2,6,10,14‐tetramethyl‐7‐(3‐methylpent‐4‐enylidene) pentadecane	348	C_25_H_48_	15.39	80.2
2,4,4‐trimethyl‐3‐hydroxymethyl‐5a‐(3‐methyl‐but‐2‐enyl)‐cyclohexene	222	C_15_H_26_O	17.47	82.7
1,3‐dioxolane, 4‐ethyl‐5‐octyl‐2,2‐bis(trifluoromethyl)‐, trans—	350	C_15_H_24_O_2_F_6_	18.58	78.4
Gamma‐tocopherol	416	C_28_H_48_O_2_	20.46	90.7
Alpha‐tocopheryl acetate	472	C_31_H_52_O_3_	20.98	88.9
Stigmasterol	412	C_29_H_48_O	21.86	72.7

### 3.3 In Vitro Antioxidant Assay

MEBCL exhibited an IC50 of 63.61 μg/mL in the DPPH assay and 130.66 μg/mL in the FRAP assay. These values are relatively comparable to those of the standard, ascorbic acid (48.10 μg/mL for the DPPH assay and 91.41 μg/mL for the FRAP assay), suggesting a strong antioxidant potential of MEBCL.

### 3.4 Acute Oral Toxicity Test

MEBCL exhibited no toxicity in the experimental animals, as no behavioral changes or mortality were observed following an oral dose of 2000 mg/kg. Consequently, the oral LD50 was determined to be greater than 2000 mg/kg. Accordingly, 200 mg/kg (one‐tenth of the highest tested dose) was designated as the low dose, and 400 mg/kg (twofold higher) was employed as the high dose for further studies.

### 3.5 In Vivo Analgesic Effects

#### 3.5.1 Acetic Acid‐Induced Writhing Method

**FIGURE 4:**
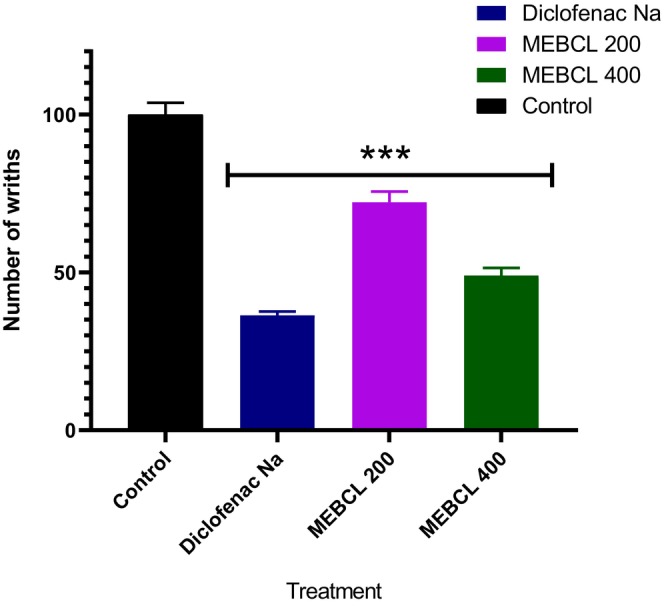
Analgesic effect of methanolic extract of *B. caudata* leaves on acetic acid‐induced writhing test. Data were presented as mean ± SEM (*n* = 5), and ***p < 0.001 was considered significant. MEBCL, methanolic extract of *B. caudata* leaves.

The average number of writhing in mice at 200 mg/kg and 400 mg/kg doses was significantly (*p* < 0.001) decreased by MEBCL, just like by the common medication diclofenac sodium (Figure 4). At doses of 200 mg/kg and 400 mg/kg, MEBCL demonstrated inhibition rates of 27.85% and 51%, respectively, while diclofenac sodium demonstrated an inhibition percentage of 63.6%.

#### 3.5.2 Formalin‐Induced Paw Licking Method

**FIGURE 5:**
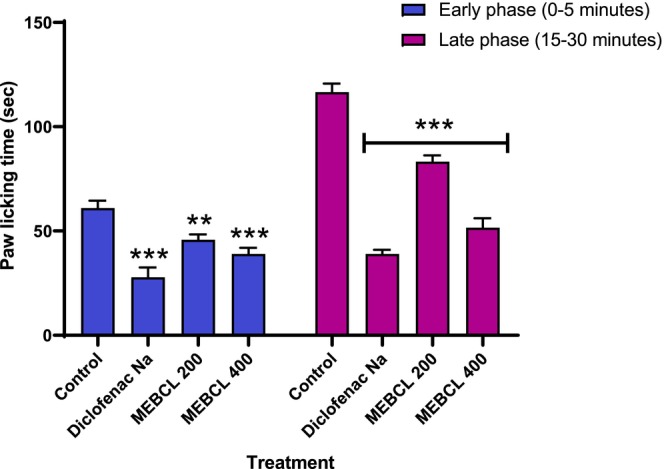
Analgesic effect of methanolic extract of *B. caudata* leaves on formalin‐induced paw‐licking test. Data were presented as mean ± SEM (*n* = 5), and ***p < 0.001 was considered significant. MEBCL, methanolic extract of *B. caudata* leaves.

As shown in Figure 5, MEBCL exhibited analgesic effects in the formalin‐induced paw‐licking test. Oral administration of MEBCL (200 and 400 mg/kg) and diclofenac sodium led to a significant (*p* < 0.001, except for MEBCL 200 mg/kg (*p* < 0.01)) reduction in licking time during the first phase, with inhibition percentages of 24.92%, 36.07%, and 54.43% compared to the control. Similarly, in the second phase, these treatments (*p* < 0.001) also reduced licking duration, achieving inhibition rates of 27.4%, 54.97%, and 65.97%, respectively, in comparison to the control group.

### 3.6 In Vivo Anxiolytic Effects

#### 3.6.1 Elevated Plus‐Maze Test

**FIGURE 6:**
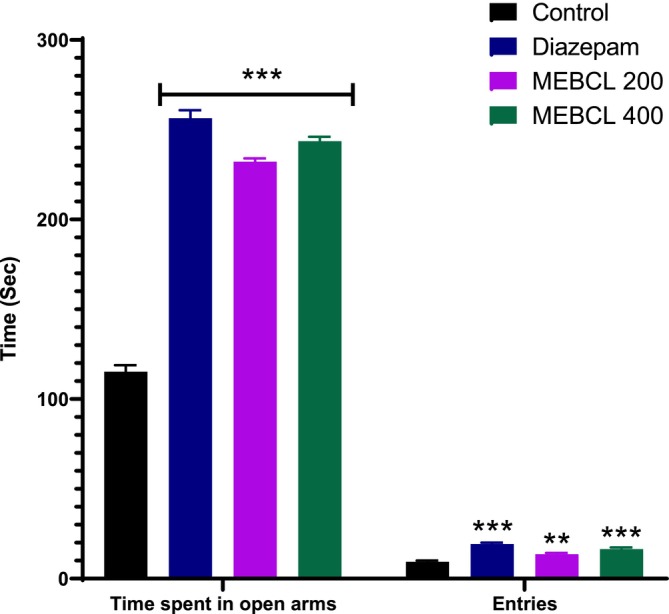
Anxiolytic effect of methanolic extract of *B. caudata* leaves on the elevated plus‐maze test. Data were presented as mean ± SEM (*n* = 5), **p < 0.01 and ***p < 0.001 were considered significant. MEBCL, methanolic extract of *B. caudata* leaves.

Figure 6 illustrates that MEBCL at both doses significantly increased both the number of entries and the duration spent in the open arms. The mice that received diazepam (1 mg/kg) also showed a higher frequency of entries and a significantly (*p* < 0.001) longer stay in the open arms.

#### 3.6.2 Hole‐Board Test

**FIGURE 7:**
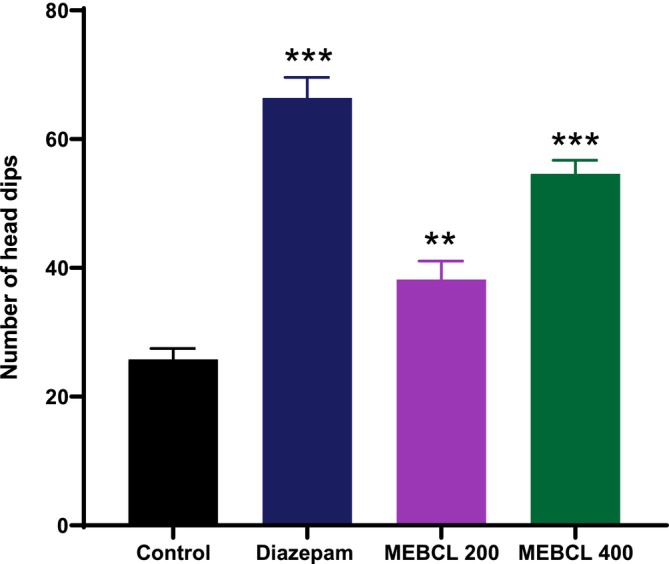
Anxiolytic effect of methanolic extract of *B. caudata* leaves on the hole board test. Data were presented as mean ± SEM (*n* = 5), **p < 0.01, and ***p < 0.001 was considered significant. MEBCL, methanolic extract of *B. caudata* leaves.

The anxiolytic activity of MEBCL was further evident in this test, as both 200 and 400 mg/kg doses significantly enhanced hole‐poking behavior, which correlates with reduced anxiety (Figure 7). Furthermore, mice in the diazepam‐treated group (1 mg/kg) displayed a significantly (*p* < 0.001) higher number of head dips than those in the control group.

### 3.7 In Vivo Anti‐Depressant Effects

#### 3.7.1 Forced Swimming Test

**FIGURE 8:**
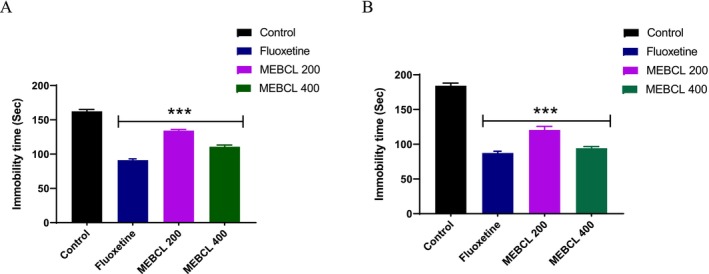
Antidepressant effect of methanolic extract of *B. caudata* leaves on (A) forced swimming test and (B) tail suspension test. Data were presented as mean ± SEM (*n* = 5), and ***p < 0.001 was considered significant. MEBCL, methanolic extract of *B. caudata* leaves.

In comparison to the control, MEBCL at doses of 200 and 400 mg/kg substantially lowered immobility time during FST in a dose‐dependent manner (Figure 8A). Mice's immobility time was significantly decreased by the standard medication, fluoxetine.

#### 3.7.2 Tail Suspension Test

Similarly, in the FST, both MEBCL doses (200 and 400 mg/kg) markedly decreased immobility time in TST relative to the control (Figure 8B), indicating its antidepressant effects.

### 3.8 Molecular Docking Analysis

The molecular docking analysis revealed that phytochemicals from MEBCL had a high binding affinity for the selected drug targets involved in oxidative stress, pain, anxiety, and depression. Protein‐ligand affinity results from molecular docking investigations are described in Table 4.

**TABLE 4:** Binding energies (kcal/mol) of phytochemicals from the methanolic extract of *B. caudata* leaves against key drug targets associated with oxidative stress, pain, anxiety, and depression.

Name	Antioxidant	Analgesic	Anxiolytic	Antidepressant
	1DGH	5IKR	6X3W	5I6X
Neophytadiene	−6.7	−6.7	−5.4	−6.8
6‐hydroxy‐4,4,7a‐trimethyl‐5,6,7,7a‐tetrahydrobenzofuran‐2(4H)‐one	−6.1	−5.6	−4.4	−7.1
Alpha‐tocopheryl acetate	−8.5	−8.2	−5.2	**−10.1**
Gamma‐tocopherol	−8.3	**−8.4**	−4.7	−9.5
2,4,4‐Trimethyl‐3‐hydroxymethyl‐5a‐(3‐methyl‐but‐2‐enyl)‐cyclohexene	−6.7	−6.5	−4.1	−7.8
Methyl 25‐methylheptacosanoate	−5.8	−6.4	−3.2	−7
Methyl 11‐methyldodecanoate	−5.9	−6.1	−4	−6.7
Phytol	−7.1	−6.3	−5.5	−7.1
Stigmasterol	**−9.1**	−6.3	**−6.6**	−9.8
11,14,17‐eicosatrienoic acid, methyl ester	−6.7	−7.1	−4.3	−7.1
Methyl 9‐cis,11‐trans‐octadecadienoate	−6.6	−6.8	−4.9	−6.9
Phytyl, 2‐methylbutanoate	−6.6	−7.5	−3.8	−7.8
2,6,10,14‐tetramethyl‐7‐(3‐methylpent‐4‐enylidene) pentadecane	−6.9	−6.3	−3.8	−7.7
1,3‐dioxolane, 4‐ethyl‐5‐octyl‐2,2‐bis(trifluoromethyl)‐, trans—	−6.8	−6.6	−4.6	−8.5
Standard (ascorbic acid/diclofenac/diazepam/fluoxetine)	−5.8	−8.2	−6.7	−9.2

*Note:* Top‐docked compound with highest binding affinities against each protein is presented in bold.

#### 3.8.1 Molecular Docking for Antioxidant Activity

**FIGURE 9:**
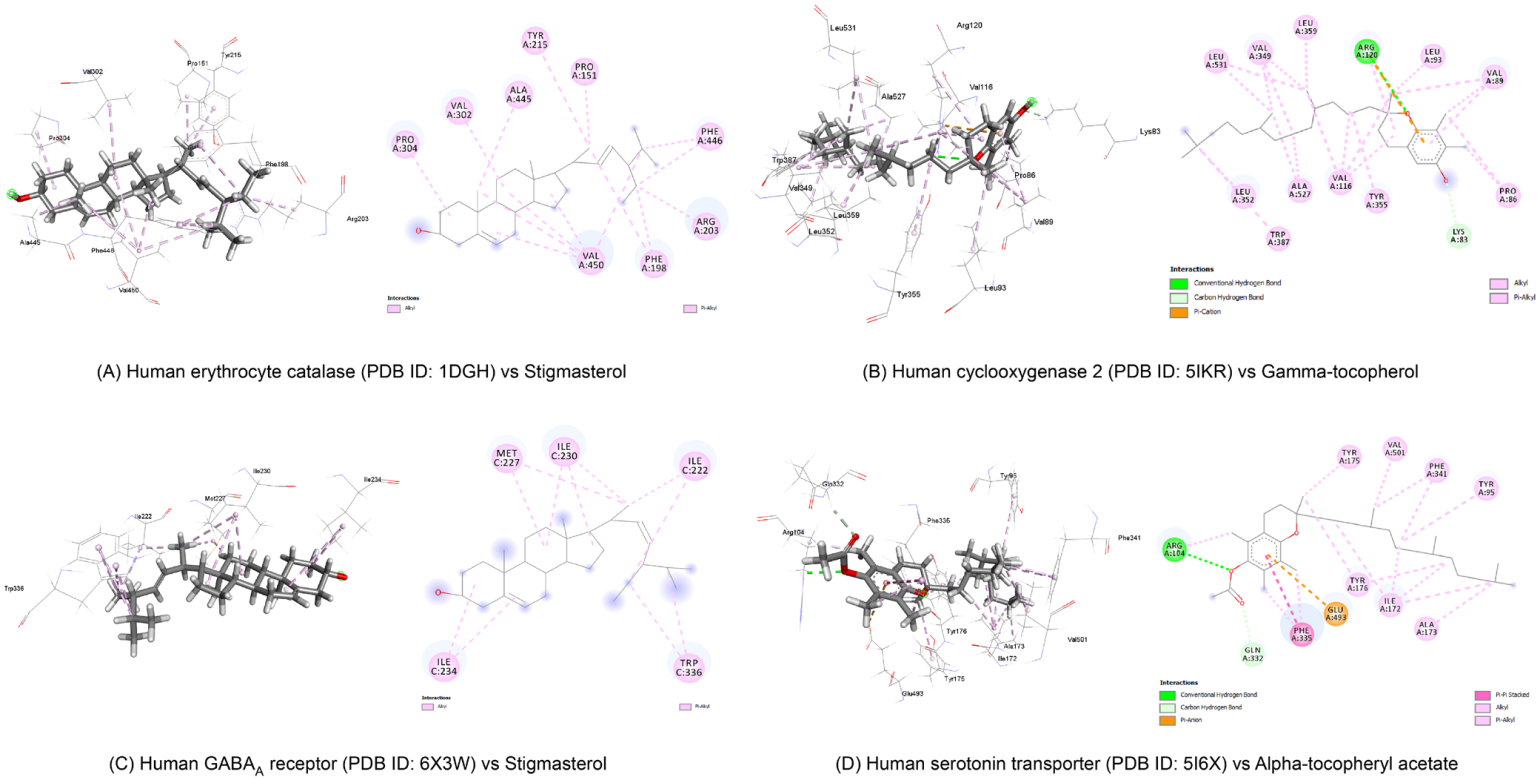
Representation of interactions of top‐docked phytochemical with each drug target protein.

The phytochemicals in MEBCL demonstrated binding affinities ranging from −9.1 to −5.8 kcal/mol against human erythrocyte catalase. Among them, stigmasterol exhibited the strongest binding affinity at −9.1 kcal/mol, exceeding the standard drug, ascorbic acid (−5.8 kcal/mol). Stigmasterol formed five alkyl bonds with Val‐450 and one alkyl bond each with Pro‐151, Arg‐203, Val‐302, Pro‐304, and Ala‐445. Additionally, it established two pi‐alkyl interactions with Phe‐198 and Phe‐446, along with a single pi‐alkyl bond with Tyr‐215 (Figure 9A).

#### 3.8.2 Molecular Docking for Analgesic Activity

COX‐2 was selected to assess the analgesic potential of MEBCL. Phytochemicals of MEBCL showed notable binding affinities (−8.4 to −5.6 kcal/mol) for COX‐2. The top‐docked compound, Gamma‐tocopherol, exhibited −8.4 kcal/mol. It formed two hydrogen bonds, one with Arg‐120 (conventional hydrogen bond) and another with Lys‐83 (carbon‐hydrogen bond). Additionally, it established a total of 19 alkyl bonds, including four with Val‐116, three with Leu‐349, two each with Pro‐86, Val‐89, Ala‐527, and Leu‐531, and one each with Leu‐93, Arg‐120, Leu‐352, and Leu‐359. A pi‐cation interaction was also observed with Arg‐120 (Figure 9B).

#### 3.8.3 Molecular Docking for Anxiolytic Activity

The GABA_A_ receptor α1‐β2‐γ2 subtype was selected to evaluate the anxiolytic potential of MEBCL phytochemicals. The binding affinities of these phytochemicals for the GABA_A_ receptor ranged from −6.6 to −3.2 kcal/mol. Stigmasterol showed the highest binding affinity (−6.6 kcal/mol), which was nearly equivalent to that of the reference drug diazepam (−6.7 kcal/mol). Stigmasterol formed nine alkyl bonds—three with Ile‐230, two with Met‐227, two with Ile‐234, one with Ile‐222, and one with Ile‐227. Additionally, it established four pi‐alkyl interactions with Trp‐336 (Figure 9C).

#### 3.8.4 Molecular Docking for Antidepressant Activity

The human serotonin transporter was selected as the target to assess the antidepressant potential of MEBCL. The binding affinities of MEBCL phytochemicals against the serotonin transporter ranged from −10.1 to −6.7 kcal/mol. Among them, alpha‐tocopheryl acetate showed the highest binding affinity at −10.1 kcal/mol, surpassing the standard antidepressant fluoxetine (−9.2 kcal/mol). Alpha‐tocopheryl acetate formed two hydrogen bonds, one with Arg‐104 (conventional hydrogen bond) and another with Glu‐332 (carbon‐hydrogen bond). The compound also established four alkyl bonds with Ile‐172 and one each with Arg‐104, Ala‐173, and Val‐501. Furthermore, it created eight pi‐alkyl interactions: two with Tyr‐176, two with Phe‐335, two with Phe‐341, and one each with Tyr‐95 and Tyr‐175. Interactions were observed with Glu‐493 (pi‐anion interaction) and Phe‐335 (pi‐pi stacked interaction) (Figure 9D).

### 3.9 ADMET Investigations

ADMET analysis was conducted to assess the pharmacokinetic properties and toxicity profiles of the compounds identified in MEBCL via GC–MS analysis (Table 5). All phytochemicals identified in MEBCL satisfied Lipinski's Rule of Five, indicating favorable drug‐likeness and structural suitability for further pharmacokinetic evaluations. This foundational criterion supports their potential as orally active drug candidates. Consistently, all compounds were predicted to be absorbed through the human intestinal tract, reinforcing their feasibility for oral administration. Moreover, the ability of all compounds to penetrate the blood–brain barrier (BBB) is particularly encouraging for targeting central nervous system (CNS) disorders or for exploring their neuroprotective potential. Regarding metabolism, none of the compounds were predicted to inhibit CYP3A4, suggesting a lower risk of drug–drug interactions at this enzymatic site. However, Stigmasterol, Alpha‐tocopheryl acetate, and Gamma‐tocopherol were identified as CYP3A4 substrates, indicating they may be metabolized more rapidly and could potentially require dose adjustments or co‐administration with metabolic enzyme modulators. In terms of safety, most compounds demonstrated a favorable toxicological profile, being nonmutagenic (AMES test), noncarcinogenic, and free from predicted liver injury (types I and II). Exceptions include 6‐hydroxy‐4,4,7a‐trimethyl‐5,6,7,7a‐tetrahydrobenzofuran‐2(4H)‐one and 1,3‐dioxolane, 4‐ethyl‐5‐octyl‐2,2‐bis(trifluoromethyl)‐, trans‐, which showed potential hepatotoxicity, suggesting the need for caution during long‐term or high‐dose use. Finally, the maximum tolerated dose (MTD) values ranged from 0.26–2.39 log mg/kg/day, with methyl 25‐methylheptacosanoate demonstrating the highest tolerance and 2,4,4‐trimethyl‐3‐hydroxymethyl‐5a‐(3‐methyl‐but‐2‐enyl)‐cyclohexene the lowest. These differences highlight variability in dose sensitivity, which should be considered during future formulation and dosing strategies (Figure 1).

**TABLE 5:** *In silico* ADMET study of phytochemicals from the methanolic extract of *B. caudata* leaves.

Name	Lipinski rule of five	Human intestinal absorption	Human oral bioavailability 50%	Blood–brain barrier	CYP 3A4 inhibitor	CYP 3A4 substrate	Half‐Life of drug	Liver injury I	Liver injury II	AMES mutagenesis	Carcinogenesis	Maximum tolerated dose
Phytol	Yes	Absorbed	Non‐Bioavailable	Penetrable	Non‐Inhibitor	Non‐Substrate	> = 3hs	Safe	Safe	Safe	Safe	0.92
2,6,10,14‐Tetramethyl‐7‐(3‐methylpent‐4‐enylidene) pentadecane	Yes	Absorbed	Non‐Bioavailable	Penetrable	Non‐Inhibitor	Non‐Substrate	> = 3hs	Safe	Safe	Safe	Safe	0.45
Neophytadiene	Yes	Absorbed	Bioavailable	Penetrable	Non‐Inhibitor	Non‐Substrate	> = 3hs	Safe	Safe	Safe	Safe	0.66
Methyl 11‐methyldodecanoate	Yes	Absorbed	Non‐Bioavailable	Penetrable	Non‐Inhibitor	Non‐Substrate	> = 3hs	Safe	Safe	Safe	Safe	1.81
11,14,17‐Eicosatrienoic Acid, methyl ester	Yes	Absorbed	Non‐Bioavailable	Penetrable	Non‐Inhibitor	Non‐Substrate	> = 3hs	Safe	Safe	Safe	Safe	1.74
Stigmasterol	Yes	Absorbed	Bioavailable	Penetrable	Non‐Inhibitor	Substrate	< 3hs	Safe	Safe	Safe	Safe	1.75
6‐Hydroxy‐4,4,7a‐trimethyl‐5,6,7,7a‐tetrahydrobenzofuran‐2(4H)‐one	Yes	Absorbed	Bioavailable	Penetrable	Non‐Inhibitor	Non‐Substrate	< 3hs	Safe	Toxic	Safe	Safe	0.34
Alpha‐tocopheryl acetate	Yes	Absorbed	Non‐Bioavailable	Penetrable	Non‐Inhibitor	Substrate	< 3hs	Safe	Safe	Safe	Safe	1.23
Gamma‐tocopherol	Yes	Absorbed	Non‐Bioavailable	Penetrable	Non‐Inhibitor	Substrate	< 3hs	Safe	Safe	Safe	Safe	0.34
2,4,4‐Trimethyl‐3‐hydroxymethyl‐5a‐(3‐methyl‐but‐2‐enyl)‐cyclohexene	Yes	Absorbed	Bioavailable	Penetrable	Non‐Inhibitor	Non‐Substrate	< 3hs	Safe	Safe	Safe	Safe	0.26
Phytyl, 2‐methylbutanoate	Yes	Absorbed	Non‐Bioavailable	Penetrable	Non‐Inhibitor	Non‐Substrate	< 3hs	Safe	Safe	Safe	Safe	1.27
Methyl 25‐methylheptacosanoate	Yes	Absorbed	Non‐Bioavailable	Penetrable	Non‐Inhibitor	Non‐Substrate	> = 3hs	Safe	Safe	Safe	Safe	2.39
Methyl 9‐Cis,11‐Trans‐Octadecadienoate	Yes	Absorbed	Non‐Bioavailable	Penetrable	Non‐Inhibitor	Non‐Substrate	> = 3hs	Safe	Safe	Safe	Safe	1.97
1,3‐Dioxolane, 4‐ethyl‐5‐octyl‐2,2‐bis(trifluoromethyl)‐, trans‐	Yes	Absorbed	Bioavailable	Penetrable	Non‐Inhibitor	Non‐Substrate	< 3hs	Safe	Toxic	Safe	Safe	0.65

## 4 Discussion

UFPs hold significant promise in addressing global food insecurity and malnutrition. They also represent a valuable reservoir of novel bioactive compounds. Despite this, many UFPs remain underexplored, limiting the realization of their full potential (Rahayu et al. 2024). In this study, we focused on the MEBCL to identify its bioactive constituents and assess its biological activities, offering new insights into its potential as a functional food.

A number of diseases are largely caused by oxidative stress, which occurs when reactive oxygen species (ROS) and the body's antioxidant defense systems are out of balance. Antioxidants counteract this process by preventing or slowing down oxidation, primarily through the inhibition of the initiation and spread of oxidative chain reactions (Halliwell 2024). Due to their ability to protect both food and pharmaceutical products from oxidative damage, as well as safeguard the body from oxidative stress‐related disorders, antioxidants have attracted considerable attention. The DPPH as well as FRAP assays are among the most common in vitro techniques for determining the antioxidant potential of plant‐based samples (Gulcin 2020). DPPH and FRAP are primarily electron transfer‐based assays that evaluate an antioxidant's capacity to reduce an oxidant, leading to a measurable color change proportional to the antioxidant concentration in the sample (Dudonne et al. 2009). The DPPH assay relies on the scavenging activity of antioxidants against the DPPH• radical, where the unpaired electron on the nitrogen atom is neutralized through hydrogen donation, forming the corresponding hydrazine. In contrast, the FRAP assay measures the ability of antioxidants to reduce Fe^3+^ to Fe^2+^, which then complexes with a ligand to produce a strong navy‐blue coloration (Abuelizz et al. 2020; Kiziltas et al. 2022). The antioxidant potential of MEBCL was confirmed through DPPH and FRAP assays, with IC_50_ values of 63.61 μg/mL and 130.66 μg/mL, respectively. Human erythrocyte catalase, a crucial antioxidant enzyme, neutralizes hydrogen peroxide into water and oxygen, safeguarding cells from oxidative stress induced by ROS (Bukowska et al. 2000). Molecular docking analysis revealed that MEBCL phytochemicals, including stigmasterol (−9.1 kcal/mol), gamma‐tocopherol (−8.5 kcal/mol), and alpha‐tocopheryl acetate (−8.3 kcal/mol), exhibited strong binding affinity for this enzyme. These compounds may enhance catalase function by improving its stability, boosting its enzymatic activity, and preventing its degradation. Thus, both in vitro as well as *in silico* investigations suggest that MEBCL phytochemicals hold promise as potent antioxidants.

In the acetic acid‐induced writhing method, pain originating in the peripheral region is triggered by acetic acid, causing abdominal contractions. The production of prostaglandins, aided by enzymes such as cyclooxygenase‐II, is essential in this process, leading to increased pain sensitivity at the sensory nerve endings (Mahnashi et al. 2022). Pain was assessed by counting the number of writhing events, with an increase in writhing indicating heightened pain perception. In our study, MEBCL at both 200 and 400 mg/kg doses significantly reduced the frequency of writhing, demonstrating its analgesic effects. Another commonly used test for analgesic evaluation is the formalin‐induced paw‐licking test, which is a persistent pain model and produces a biphasic nociceptive response. The initial stage, which starts right after the formalin injection and lasts for roughly 10 min, is mediated by pain fibers, particularly C fibers. After the injection, the second phase starts about 15 min later and lasts for about 60 min, being primarily driven by peripheral inflammation (Deng et al. 2021). Both doses of MEBCL significantly reduced paw licking in both phases, indicating its dual action through peripheral mechanisms and anti‐inflammatory effects. The molecular docking study revealed that MEBCL compounds, such as gamma‐tocopherol (−8.4 kcal/mol) and alpha‐tocopheryl acetate (−8.2 kcal/mol), exhibited binding affinities similar to the standard drug, diclofenac (−8.2 kcal/mol). Therefore, COX‐2 inhibition may be a potential mechanism responsible for the analgesic effects of *B. caudata* leaves.

The anxiolytic effects of MEBCL were assessed using two well‐known behavioral models: the EPM and HBT. The EPM test is widely used for testing anxiety behavior in animals, where open and elevated sections of the maze induce fear and anxiety, prompting mice to avoid these areas and spend more time in the closed (safer) arms. Treatments for anxiety promote exploratory behavior, which lengthens the time spent and increases the quantity of entries in the open arms (Shah et al. 2022). In our study, MEBCL substantially enhanced these parameters, indicating its anxiolytic effects. In the HBT, anxiety levels are evaluated by counting the head dips, with anxiolytic drugs increasing this behavior (Goni et al. 2021). MEBCL (200 and 400 mg/kg) significantly raised the head‐dip counts compared to the control group, further confirming its anxiolytic properties. Many commonly prescribed anxiolytics, such as benzodiazepines and barbiturates, function by modulating GABAergic activity. Our molecular docking studies indicated that the phytochemicals in MEBCL exhibit strong binding affinities for the GABA_A_ receptor, with stigmasterol being the top‐docked compound showing a binding affinity (−6.6 kcal/mol) similar to that of the standard anxiolytic drug, diazepam (−6.7 kcal/mol). A study by Karim et al. confirmed that stigmasterol has in vivo anxiolytic effects through positive modulation of the GABAergic system, aligning with our findings (Karim et al. 2021). Therefore, the presence of stigmasterol, along with other CNS‐active compounds, likely contributes to the anxiolytic effects observed in *B. caudata* leaves.

FST and TST are two inexpensive and easy‐to‐perform methods utilized to determine the effectiveness of antidepressant agents in mice. One advantage of TST over other behavioral tests is its ability to minimize the influence of motor impairments and hypothermia on results. Both FST and TST rely on measuring immobility, which is considered an indicator of depressive‐like behavior (Alegiry et al. 2022). When subjected to inescapable stressful conditions, animals exhibit prolonged immobility, signifying despair or an inability to adapt. This behavior closely mirrors depressive symptoms in humans. The administration of an antidepressant reduces immobility time, indicating its therapeutic potential (Shah et al. 2022). In this study, MEBCL significantly decreased the immobility duration in both test models at both tested doses compared to the control group. The serotonergic system is essential for controlling appetite, sleep, mood, and emotions, making it a key target for antidepressant therapies. The serotonin transporter is a primary target of many clinically approved antidepressants (Schloss and Williams 1998). Molecular docking analysis demonstrated that phytochemicals in MEBCL exhibit strong binding affinities for the serotonin transporter protein. Alpha‐tocopheryl acetate (−10.1 kcal/mol), stigmasterol (−9.8 kcal/mol), and gamma‐tocopherol (−9.5 kcal/mol) showed higher binding affinities than fluoxetine (−9.2 kcal/mol), a standard antidepressant drug. These compounds are capable of crossing the blood–brain barrier and may exert significant antidepressant effects.

The pathogenesis of anxiety and depression is often associated with imbalances in brain neurotransmitter levels. However, a growing body of evidence suggests that oxidative stress plays a significant role in the development of these neuropsychiatric disorders (Black et al. 2017). Oxidative stress induces lipid peroxidation in the brain, leading to reduced membrane fluidity and damage to membrane proteins, which ultimately disrupts critical signaling pathways and impairs normal brain function. Elevated oxidative stress markers have also been observed in individuals suffering from anxiety and depression. Although treatment with antidepressants has shown improvement in oxidative stress markers, diazepam (a widely used anxiolytic) fails to completely reverse oxidative stress‐induced anxiety (Bouayed et al. 2009; Smaga et al. 2015). Therefore, a therapeutic strategy that combines antioxidant, anxiolytic, and antidepressant properties may offer enhanced benefits. In this context, MEBCL holds promise as a potential candidate for managing both neurotransmitter‐related and oxidative stress‐associated anxiety and depression.

FT‐IR and GC–MS analysis revealed that MEBCL contains bioactive compounds like Vitamin E, flavonoids, terpenes, fatty acid esters, and sterols. Research has previously highlighted the pharmacological benefits of these compounds. For instance, gamma‐tocopherol (Vitamin E) is recognized for its potent antioxidant properties and its ability to inhibit inflammatory drug targets such as COX‐2, thereby exhibiting anti‐inflammatory effects (Jiang et al. 2022). Alpha‐tocopheryl acetate, a salt form of Vitamin E, is converted into alpha‐tocopherol in the body and also demonstrates antioxidant and analgesic effects (de Azevedo Filho et al. 2025; Meulmeester et al. 2022). Clinical studies have shown that alpha‐tocopherol can help alleviate anxiety and depression in humans (Lee et al. 2022). Additionally, phytol, a diterpene alcohol, shows antioxidant properties due to the allylic alcohol group and has exhibited in vivo analgesic and anxiolytic effects (M. T. Islam et al. 2018). Another diterpene identified, neophytadiene, demonstrated significant anxiolytic and antidepressant effects in male CD‐1 mice (Gonzalez‐Rivera et al. 2023). Another compound, stigmasterol, a phytosterol, demonstrates both in vitro and in vivo antioxidant properties, as well as neuroprotective and anxiolytic actions (Bakrim et al. 2022). These bioactive compounds could contribute to the effects observed by MEBCL in our study. ADMET analysis revealed that these phytochemicals conform to Lipinski's rule of five, which indicates favorable drug‐like properties. Furthermore, their safety profile indicates that they may potentially qualify as drug candidates.

## 5 Limitations and Future Perspectives

The present investigation has several limitations that should be acknowledged. First, phytochemical characterization of MEBCL was carried out solely using GC–MS, which is restricted to detecting volatile and semi‐volatile compounds. Future studies should employ LC–MS, along with isolation and structural elucidation of pure bioactive constituents, to achieve a more comprehensive chemical profile. Second, the pharmacological activities were assessed primarily through observational methods; more detailed investigations at the molecular and mechanistic levels are needed to identify the precise pathways responsible for the observed biological effects. Third, the study evaluated only two doses (200 and 400 mg/kg). A comprehensive dose‐dependent study is required to better establish the extract's safety margin and therapeutic efficacy. Finally, the experiments were conducted solely in mouse models. Future research should expand to other rodent models and eventually progress to primate and human clinical studies to validate translational potential.

## 6 Conclusion

Methanolic extract of *B. caudata* leaves was analyzed using GC–MS, which revealed 14 compounds, including two forms of vitamin E. MEBCL exhibited in vitro antioxidant effects and in vivo analgesic, anxiolytic, and antidepressant activities. Molecular docking and ADMET studies revealed that the compounds from MEBCL demonstrated strong binding affinity toward key drug target proteins related to oxidative stress, pain, anxiety, and depression, while also showing a favorable pharmacokinetic and safety profile. This study demonstrated *B. caudata* leaves' potential as a promising source of food and medicine, laying the groundwork for further investigations. Additional experimental validation is required in order to fully comprehend the mechanisms of action and safety profile of *B. caudata* leaves.’

## Author Contributions

**Md. Liakot Ali:** conceptualization (equal), data curation (equal), formal analysis (equal), investigation (equal), methodology (equal), visualization (equal), writing – original draft (equal). **Md. Ajib Jaber:** investigation (equal), writing – review and editing (equal). **Zihanul Hasan:** investigation (equal), writing – review and editing (equal). **Nawreen Monir Proma:** project administration (equal), writing – review and editing (equal). **Neamul Hoque:** investigation (equal), writing – review and editing (equal). **Md. Tashrif Rahman Tipu:** investigation (equal), writing – review and editing (equal). **Kutub Uddin Ahamed:** investigation (equal), writing – review and editing (equal). **Bakul Akter:** investigation (equal), writing – review and editing (equal). **Bibi Humayra Khanam:** investigation (equal), writing – review and editing (equal). **Mohammed Kamrul Hossain:** conceptualization (equal), methodology (equal), supervision (equal), writing – review and editing (equal).

## Ethics Statement

The Animal Ethics Review Board (AERB), Faculty of Biological Sciences, University of Chittagong, approved this study using approval form number AERB‐FBSCU‐20250622‐(1).

## Conflicts of Interest

The authors declare no conflicts of interest.

## Supporting information


**Data S1:** Supporting Information.

## Data Availability

The data that support the findings of this study are available on request from the corresponding author.

## References

[fsn370953-bib-0001] Abuelizz, H. A. , E. Anouar , M. Marzouk , H. A. A. Taie , A. Ahudhaif , and R. Al‐Salahi . 2020. “DFT Study and Radical Scavenging Activity of 2‐Phenoxypyridotriazolo Pyrimidines by DPPH, ABTS, FRAP and Reducing Power Capacity.” Chemical Papers 74, no. 9: 2893–2899.

[fsn370953-bib-0002] Akter, K.‐M. , H.‐J. Kim , A. A. K. Khalil , et al. 2018. “Inner Morphological and Chemical Differentiation of Boehmeria Species.” Journal of Natural Medicines 72, no. 2: 409–423. 29260412 10.1007/s11418-017-1164-8

[fsn370953-bib-0003] Alegiry, M. H. , A. El Omri , A. A. Bayoumi , M. Y. Alomar , I. A. Rather , and J. S. M. Sabir . 2022. “Antidepressant‐Like Effect of Traditional Medicinal Plant *Carthamus Tinctorius* in Mice Model Through Neuro‐Behavioral Tests and Transcriptomic Approach.” Applied Sciences 12, no. 11: 5594.

[fsn370953-bib-0004] Ali, M. L. , J. N. Meem , N. Hoque , et al. 2024. “GC‐MS Analysis, Neuropharmacological and Antidiarrheal Activities of the Acetone Extract of *Najas Gracillima* Seaweed: In Vivo and In Silico Study.” Chemistry & Biodiversity 22, no. 5: e202402303. 10.1002/cbdv.202402303 39714997

[fsn370953-bib-0005] Ali, M. L. , F. Noushin , Q. A. Sadia , et al. 2024. “Spices and Culinary Herbs for the Prevention and Treatment of Breast Cancer: A Comprehensive Review With Mechanistic Insights.” Cancer Pathogenesis and Therapy 3, no. 3: 197–214. 10.1016/j.cpt.2024.07.003. 40458304 PMC12126743

[fsn370953-bib-0006] Ayanaw, M. A. , J. S. Yesuf , and E. M. Birru . 2023. “Evaluation of Analgesic and Anti‐Inflammatory Activities of Methanolic Leaf and Root Extracts of Gomphocarpus Purpurascens A. Rich (Asclepiadaceae) in Mice.” Journal of Experimental Pharmacology 15: 1–11. 36643970 10.2147/JEP.S361194 PMC9838122

[fsn370953-bib-0007] Azam, F. M. S. , A. Biswas , A. Mannan , N. A. Afsana , R. Jahan , and M. Rahmatullah . 2014. “Are Famine Food Plants Also Ethnomedicinal Plants? An Ethnomedicinal Appraisal of Famine Food Plants of Two Districts of Bangladesh.” Evidence‐Based Complementary and Alternative Medicine 2014, no. 1: 741712. 24701245 10.1155/2014/741712 PMC3950545

[fsn370953-bib-0008] Bakrim, S. , N. Benkhaira , I. Bourais , et al. 2022. “Health Benefits and Pharmacological Properties of Stigmasterol.” Antioxidants 11, no. 10: 1912. 36290632 10.3390/antiox11101912 PMC9598710

[fsn370953-bib-0009] Black, C. N. , M. Bot , P. G. Scheffer , and B. Penninx . 2017. “Oxidative Stress in Major Depressive and Anxiety Disorders, and the Association With Antidepressant Use; Results From a Large Adult Cohort.” Psychological Medicine 47, no. 5: 936–948. 27928978 10.1017/S0033291716002828

[fsn370953-bib-0010] Bouayed, J. , H. Rammal , and R. Soulimani . 2009. “Oxidative Stress and Anxiety: Relationship and Cellular Pathways.” Oxidative Medicine and Cellular Longevity 2, no. 2: 63–67. 20357926 10.4161/oxim.2.2.7944 PMC2763246

[fsn370953-bib-0011] Bukowska, B. , A. Chajdys , W. Duda , and P. Duchnowicz . 2000. “Catalase Activity in Human Erythrocytes: Effect of Phenoxyherbicides and Their Metabolites.” Cell Biology International 24, no. 10: 705–711. 11023648 10.1006/cbir.2000.0553

[fsn370953-bib-0012] Chung, B. S. , and M. K. Shin . 1998. Illustrated Dictionary of Folk Medicine (Crude Drugs), 820–822. Younglimsa.

[fsn370953-bib-0013] da Cruz Alves, T. , V. F. Kinupp , B. A. F. de Mendonça , and T. B. Breier . 2024. “Non‐Conventional Food Plants (Plantas Alimentícias Não Convencionais (PANC)) of the Petrópolis–Teresópolis Crossing, Serra dos Órgãos National Park, Rio de Janeiro, Brazil.” Wild 1, no. 1: 17–29.

[fsn370953-bib-0014] Daina, A. , O. Michielin , and V. Zoete . 2017. “SwissADME: A Free Web Tool to Evaluate Pharmacokinetics, Drug‐Likeness and Medicinal Chemistry Friendliness of Small Molecules.” Scientific Reports 7, no. 1: 42717. 28256516 10.1038/srep42717 PMC5335600

[fsn370953-bib-0015] Dallakyan, S. , and A. J. Olson . 2015. “Small‐Molecule Library Screening by Docking With PyRx.” In Chemical Biology: Methods and Protocols, 1263, 243–250. Springer. 10.1007/978-1-4939-2269-7_19 25618350

[fsn370953-bib-0016] de Azevedo Filho, F. A. S. , Y. M. C. de Castro , M. P. Cerqueira , T. A. Rodrigues , Y. Ali‐Abdouni , and P. M. d. M. B. Fucs . 2025. “Use of Ascorbic Acid (Vitamin C) and Alpha Tocopherol (Vitamin E) as Adjuvants in the Treatment of Neuropathic Pain.” BrJP 8: e20250005.

[fsn370953-bib-0017] de Paiva, P. P. , F. R. Nonato , A. L. T. G. Ruiz , et al. 2020. “An Ethanolic Extract of Boehmeria caudata Aerial Parts Displays Anti‐Inflammatory and Anti‐Tumor Activities.” Planta Medica International Open 7, no. 1: e17–e25.

[fsn370953-bib-0018] de Paiva, P. P. , J. H. B. Nunes , F. R. Nonato , et al. 2020. “In Silico, In Vitro, and In Vivo Antitumor and Anti‐Inflammatory Evaluation of a Standardized Alkaloid‐Enriched Fraction Obtained From Boehmeria caudata Sw. Aerial Parts.” Molecules 25, no. 17: 4018. 32899132 10.3390/molecules25174018 PMC7504783

[fsn370953-bib-0019] Deng, J. , J. Han , J. Chen , et al. 2021. “Comparison of Analgesic Activities of Aconitine in Different Mice Pain Models.” PLoS One 16, no. 4: e0249276. 33793632 10.1371/journal.pone.0249276 PMC8016268

[fsn370953-bib-0020] Dudonne, S. , X. Vitrac , P. Coutiere , M. Woillez , and J.‐M. Mérillon . 2009. “Comparative Study of Antioxidant Properties and Total Phenolic Content of 30 Plant Extracts of Industrial Interest Using DPPH, ABTS, FRAP, SOD, and ORAC Assays.” Journal of Agricultural and Food Chemistry 57, no. 5: 1768–1774. 19199445 10.1021/jf803011r

[fsn370953-bib-0021] Ferreira Júnior, W. S. , L. Z. Campos , and P. M. de Medeiros . 2021. “Unconventional Food Plants: Food or Medicine?” In Local Food Plants of Brazil, 29–47. Springer.

[fsn370953-bib-0022] Garber, J. , and V. R. Weersing . 2010. “Comorbidity of Anxiety and Depression in Youth: Implications for Treatment and Prevention.” Clinical Psychology: Science and Practice 17, no. 4: 293–306. 21499544 10.1111/j.1468-2850.2010.01221.x PMC3074295

[fsn370953-bib-0023] Goni, O. , M. F. Khan , M. M. Rahman , et al. 2021. “Pharmacological Insights on the Antidepressant, Anxiolytic and Aphrodisiac Potentials of Aglaonema Hookerianum Schott.” Journal of Ethnopharmacology 268: 113664. 33278545 10.1016/j.jep.2020.113664

[fsn370953-bib-0024] Gonzalez‐Rivera, M. L. , J. C. Barragan‐Galvez , D. Gasca‐Martínez , S. Hidalgo‐Figueroa , M. Isiordia‐Espinoza , and A. J. Alonso‐Castro . 2023. “In Vivo Neuropharmacological Effects of Neophytadiene.” Molecules 28, no. 8: 3457. 37110691 10.3390/molecules28083457 PMC10142729

[fsn370953-bib-0025] Guex, N. , and M. C. Peitsch . 1997. “SWISS‐MODEL and the Swiss‐Pdb Viewer: An Environment for Comparative Protein Modeling.” Electrophoresis 18, no. 15: 2714–2723. 9504803 10.1002/elps.1150181505

[fsn370953-bib-0026] Gulcin, İ. 2020. “Antioxidants and Antioxidant Methods: An Updated Overview.” Archives of Toxicology 94, no. 3: 651–715. 32180036 10.1007/s00204-020-02689-3

[fsn370953-bib-0027] Halliwell, B. 2024. “Understanding Mechanisms of Antioxidant Action in Health and Disease.” Nature Reviews Molecular Cell Biology 25, no. 1: 13–33. 37714962 10.1038/s41580-023-00645-4

[fsn370953-bib-0028] Hossen, M. A. , A. S. M. Ali Reza , M. B. Amin , et al. 2021. “Bioactive Metabolites of *Blumea lacera* Attenuate Anxiety and Depression in Rodents and Computer‐Aided Model.” Food Science & Nutrition 9, no. 7: 3836–3851. 34262741 10.1002/fsn3.2362 PMC8269660

[fsn370953-bib-0029] Islam, M. R. , M. T. Chowdhury , M. M. Chowdhury , et al. 2025. “Investigating the Secondary Metabolite Profile and Neuropharmacological Activities of *Ipomoea Purpurea*: A Multi‐Method Approach Using GC‐MS, In Vivo, and In Silico Techniques.” Chemistry & Biodiversity: e202500560. 40263108 10.1002/cbdv.202500560

[fsn370953-bib-0030] Islam, M. T. , E. S. Ali , S. J. Uddin , et al. 2018. “Phytol: A Review of Biomedical Activities.” Food and Chemical Toxicology 121: 82–94. 30130593 10.1016/j.fct.2018.08.032

[fsn370953-bib-0031] Jiang, Q. , S. Im , J. G. Wagner , M. L. Hernandez , and D. B. Peden . 2022. “Gamma‐Tocopherol, a Major Form of Vitamin E in Diets: Insights Into Antioxidant and Anti‐Inflammatory Effects, Mechanisms, and Roles in Disease Management.” Free Radical Biology and Medicine 178: 347–359. 34896589 10.1016/j.freeradbiomed.2021.12.012 PMC8826491

[fsn370953-bib-0032] Karim, N. , I. Khan , A. Abdelhalim , S. A. Halim , A. Khan , and A. Al‐Harrasi . 2021. “Stigmasterol Can Be New Steroidal Drug for Neurological Disorders: Evidence of the GABAergic Mechanism via Receptor Modulation.” Phytomedicine 90: 153646. 34280827 10.1016/j.phymed.2021.153646

[fsn370953-bib-0033] Kim, S.‐M. , D.‐I. Shin , H.‐S. Song , S.‐K. Kim , and S.‐T. Yoon . 2006. “Geographical Distribution and Habitat Characteristics of Boehmeria in South Korea.” Korean Journal of Medicinal Crop Science 14, no. 1: 14–18.

[fsn370953-bib-0034] Kiziltas, H. , A. C. Goren , S. H. Alwasel , and İ. Gulcin . 2022. “Sahlep (Dactylorhiza Osmanica): Phytochemical Analyses by LC‐HRMS, Molecular Docking, Antioxidant Activity, and Enzyme Inhibition Profiles.” Molecules 27, no. 20: 6907. 36296499 10.3390/molecules27206907 PMC9611915

[fsn370953-bib-0035] Lee, A. R. Y. , A. Bin Tariq , G. Lau , N. W. K. Tok , W. W. S. Tam , and C. S. H. Ho . 2022. “Vitamin E, Alpha‐Tocopherol, and Its Effects on Depression and Anxiety: A Systematic Review and Meta‐Analysis.” Nutrients 14, no. 3: 656. 35277015 10.3390/nu14030656 PMC8840247

[fsn370953-bib-0036] Mahnashi, M. H. , Y. S. Alqahtani , B. A. Alyami , et al. 2022. “GC‐MS Analysis and Various in Vitro and in Vivo Pharmacological Potential of Habenaria Plantaginea Lindl.” Evidence‐Based Complementary and Alternative Medicine 2022, no. 1: 7921408. 35399645 10.1155/2022/7921408 PMC8989558

[fsn370953-bib-0037] Meulmeester, F. L. , J. Luo , L. G. Martens , K. Mills , D. van Heemst , and R. Noordam . 2022. “Antioxidant Supplementation in Oxidative Stress‐Related Diseases: What Have We Learned From Studies on Alpha‐Tocopherol?” Antioxidants 11, no. 12: 2322. 36552530 10.3390/antiox11122322 PMC9774512

[fsn370953-bib-0038] Milião, G. L. , A. P. H. De Oliveira , L. de Souza Soares , T. R. Arruda , É. N. R. Vieira , and B. R. d. C. L. Junior . 2022. “Unconventional Food Plants: Nutritional Aspects and Perspectives for Industrial Applications.” Future Foods 5: 100124.

[fsn370953-bib-0039] Myung, Y. , A. G. C. de Sá , and D. B. Ascher . 2024. “Deep‐PK: Deep Learning for Small Molecule Pharmacokinetic and Toxicity Prediction.” Nucleic Acids Research 52, no. W1: W469–W475. 38634808 10.1093/nar/gkae254 PMC11223837

[fsn370953-bib-0040] O'Boyle, N. M. , M. Banck , C. A. James , C. Morley , T. Vandermeersch , and G. R. Hutchison . 2011. “Open Babel: An Open Chemical Toolbox.” Journal of Cheminformatics 3: 1–14. 21982300 10.1186/1758-2946-3-33 PMC3198950

[fsn370953-bib-0041] Peisino, M. C. O. , M. S. Zouain , M. M. de Christo Scherer , et al. 2020. “Health‐Promoting Properties of Brazilian Unconventional Food Plants.” Waste and Biomass Valorization 11: 4691–4700.

[fsn370953-bib-0042] Perl, E. R. 2011. “Pain Mechanisms: A Commentary on Concepts and Issues.” Progress in Neurobiology 94, no. 1: 20–38. 21419824 10.1016/j.pneurobio.2011.03.001 PMC3138063

[fsn370953-bib-0043] Rahayu, Y. Y. S. , W. Sujarwo , A. S. D. Irsyam , A. Dwiartama , and D. Rosleine . 2024. “Exploring Unconventional Food Plants Used by Local Communities in a Rural Area of West Java, Indonesia: Ethnobotanical Assessment, Use Trends, and Potential for Improved Nutrition.” Journal of Ethnobiology and Ethnomedicine 20, no. 1: 68. 39030547 10.1186/s13002-024-00710-y PMC11264525

[fsn370953-bib-0044] Sany, J. T. , M. L. Ali , M. E. H. Ekram , and M. T. Ahsan . 2025. “Investigating the Therapeutic Potential of *Baccaurea Motleyana* Through an Integrated Approach Utilizing In Vitro, In Vivo, and In Silico Investigations.” Clinical Traditional Medicine and Pharmacology 6: 200197. 10.1016/j.ctmp.2025.200197.

[fsn370953-bib-0045] Schloss, P. , and D. C. Williams . 1998. “The Serotonin Transporter: A Primary Target for Antidepressant Drugs.” Journal of Psychopharmacology 12, no. 2: 115–121. 9694022 10.1177/026988119801200201

[fsn370953-bib-0046] Shah, M. S. , M. A. Tayab , A. Rahman , et al. 2022. “Anxiolytic, Antidepressant and Antioxidant Activity of the Methanol Extract of Canarium Resiniferum Leaves.” Journal of Traditional and Complementary Medicine 12, no. 6: 567–574. 36325239 10.1016/j.jtcme.2022.07.001 PMC9618395

[fsn370953-bib-0047] Singh, T. P. , G. Chauhan , R. K. Agrawal , and S. K. Mendiratta . 2019. “In Vitro Study on Antimicrobial, Antioxidant, FT‐IR and GC–MS/MS Analysis of *Piper betle* L. Leaves Extracts.” Journal of Food Measurement and Characterization 13: 466–475.

[fsn370953-bib-0048] Smaga, I. , E. Niedzielska , M. Gawlik , et al. 2015. “Oxidative Stress as an Etiological Factor and a Potential Treatment Target of Psychiatric Disorders. Part 2. Depression, Anxiety, Schizophrenia and Autism.” Pharmacological Reports 67, no. 3: 569–580. 25933971 10.1016/j.pharep.2014.12.015

[fsn370953-bib-0049] Studio, D. 2008. “Discovery Studio.” Accelrys [2.1] 420: 1–9.

[fsn370953-bib-0050] Yazbek, P. B. , P. Matta , L. F. Passero , et al. 2019. “Plants Utilized as Medicines by Residents of Quilombo da Fazenda, Núcleo Picinguaba, Ubatuba, São Paulo, Brazil: A Participatory Survey.” Journal of Ethnopharmacology 244: 112123. 31356967 10.1016/j.jep.2019.112123

