# Supplementary material for: Phytochemical and Bioactivity Profiling of Unconventional Food Plant, Boehmeria caudata Leaves: FT‐IR, GC–MS, Experimental, and In Silico Investigation
Source: Food Sci Nutr. 2025 Sep 14;13(9):e70953. doi: 10.1002/fsn3.70953 (PMC12433900; doi:10.1002/fsn3.70953)
Supplement: Supplementary file 1 — Data S1: Supporting Information. [file FSN3-13-e70953-s001.docx]

**Fourier transform-infrared (FT‑IR) spectroscopy analysis**

The FT-IR analysis of MEBCL was conducted by positioning it at the exit point of the KBr (FT-IR grade) pellet. These pellets were formed from powdered KBr, which had been pre-activated at 60°C for 24 hours, using a pellet press machine. The infrared spectra of the extract were recorded within the wavelength range of 4000–400 cm⁻¹ utilizing a Thermo Nicolet 6700 FT-IR spectrometer, equipped with a DTGS-XT-KBr detector and an Xt-KBr beam splitter assembly. Each spectrum was obtained over 32 scans with a resolution of 4.0 cm⁻¹.

**Gas chromatography-mass spectroscopy (GC-MS) analysis**

The GC-MS analysis of MEBCL was performed using a SHIMADZU GCMS-QP2020 system, integrated with an AOC-20s auto-sampler and an AOC-20i auto-injector. Separation was carried out using a capillary column (30 m length, 0.25 mm internal diameter) coated with a stationary phase comprising 5% diphenyl and 95% dimethyl polysiloxane. The injection port temperature was maintained at 220°C. The oven temperature program started at 80°C (held for 2 minutes), then increased to 150°C at a rate of 5°C per minute, and finally reached 280°C. The ion source temperature remained at 280°C. Helium was used as the carrier gas at a flow rate of 1.72 mL/min. A 4 µL sample was injected in split-less mode with a split ratio of 1:100. The GC-MS detector functioned at an ionizing energy of 45 m/z with a scan interval of 0.30 seconds. The total analysis duration was 50 minutes. Mass spectral identification was performed using the National Institute of Standards and Technology (NIST) database, comparing the obtained spectra with reference compounds in the 2008 and 2014 NIST library editions.
